# Role of Different Growth Enhancers as Alternative to In-feed Antibiotics in Poultry Industry

**DOI:** 10.3389/fvets.2021.794588

**Published:** 2022-02-11

**Authors:** Kazi Rafiq, Muhammad Tofazzal Hossain, Rokeya Ahmed, Md. Mehedi Hasan, Rejaul Islam, Md. Ismail Hossen, Sourendra Nath Shaha, Mohammad Rafiqul Islam

**Affiliations:** ^1^Department of Pharmacology, Bangladesh Agricultural University, Mymensingh, Bangladesh; ^2^Department of Microbiology and Hygiene, Bangladesh Agricultural University, Mymensingh, Bangladesh; ^3^Livestock Division, Bangladesh Agricultural Research Council, Dhaka, Bangladesh; ^4^Department of Livestock Services, Dhaka, Bangladesh

**Keywords:** growth promoters, probiotics, prebiotics, phytobiotics, spirulina, synbiotic, poultry

## Abstract

The poultry industry has grown so fast alongside the irrational use of antibiotics to maximize profit and make the production cost-effective during the last few decades. The rising and indiscriminate use of antibiotics might result in the deposition of residues in poultry food products and in the development of resistance to these drugs by microorganisms. Therefore, many diseases are becoming difficult to treat both in humans and animals. In addition, the use of low-dose antibiotics as growth enhancer results in antibiotic residues in food products, which have detrimental effects on human health. On the other hand, many studies have shown that antibiotics administered to poultry and livestock are poorly absorbed through the gut and usually excreted without metabolism. These excreted antibiotics eventually accumulate in the environment and enter the human food chain, resulting in the bioaccumulation of drug residues in the human body. In this regard, to find out alternatives is of paramount importance for the production of safe meat and egg. Therefore, in recent years, much research attention was disarticulated toward the exploration for alternatives to antibiotic as in-feed growth enhancers after its ban by the EU. As a result, probiotics, prebiotics, phytobiotics, spirulina, symbiotic, and their combination are being used more frequently in poultry production. Feed additives therefore gained popularity in poultry production by having many advantages but without any residues in poultry products. In addition, numerous studies demonstrating that such biological supplements compete with antimicrobial resistance have been conducted. Therefore, the purpose of this review article was to highlight the advantages of using biological products instead of antibiotics as poultry in-feed growth enhancers to enhance the production performance, reduce intestinal pathogenic bacteria, and maintain gut health, potentiating the immune response, safety, and wholesomeness of meat and eggs as evidence of consumer protection, as well as to improve the safety of poultry products for human consumption.

## Introduction

Antimicrobial resistance (AMR) is now widely acknowledged as one of the most important global public health threats. The main reason behind this hazard is the irrational and indiscriminate use of antimicrobial drugs in humans, livestock, and the poultry industry. The poultry industry is now considered as one of the fastest-growing subsector of agriculture and veterinary fields due to the increased consumption of meat and egg, making these easily accessible, with a relatively low cost, and rich in most essential nutrients (1). Because of the ease of usage and low cost, antibiotic growth enhancers (AGPs) have been widely used in poultry production around the world. It has changed intensive poultry production by increasing gut health and reducing subclinical infections while also promoting growth, production, and feed conversion efficiency. Antibiotics in low doses improve gut health by reducing the pathogen load and helps in the prevention of subclinical infection in poultry even in well-managed poultry farms. In addition, antibiotics have a number of advantages, including thickening of the gut, which leads to increased nutrient absorption (2). Antibiotics are now given to broiler chicken at a low dose in order to promote a faster growth (3). In contrast to developed economies, the increasing and indiscriminate use of sub-therapeutic doses of antibiotic has had a negative influence on the balance of the normal inhabitants of the gut microflora, gathering antibiotic residues in tissue as well as developing new strains of drug-resistant pathogenic bacteria by mutation or plasmid-mediated transfer (4). This resistant bacteria population enters into the human body through consumption and handling of meat and eggs contaminated with such pathogens that are resistant to antimicrobial drugs (5). Once exposed, the resistant bacteria (superbugs) colonize the intestinal tract of the hosts, and the gene coding for antibiotic resistance in these bacteria can be transferred to other bacteria in the endogenous microflora of the host species, causing a delay in the intervention of bacterial infection (6). On the other hand, destruction of beneficial microbes by antibiotics makes the birds more susceptible to the development of harmful bacteria and coccidia. Therefore, in 2005, the use of antibiotic in animal feed as growth enhancer was banned by the European Union Commission. Antibiotic resistance is being closely monitored, and the governments of many countries are considering banning antibiotic growth enhancers, resulting in a growing interest in discovering viable antibiotic replacements in both layer and broiler production (7). Meanwhile, many alternative substances have been investigated for their potential to replace antibiotic as in-feed growth enhancers. In this regard, numerous research have been carried out to look for natural agents which mimic the similar beneficial effects of growth enhancers, and a lot of researchers have replaced antibiotics with effective dietary supplements, such as probiotic, pre-biotic, phytobiotic, spirulina, symbiotic, and their combination, which are claimed to enhance growth performance and carcass weight and decrease the mortality rate (7, 8). These natural products and their combination have a positive effect on food conversion ratio (FCR), and their residues have not been stored in poultry product and are proven safe. Besides these, these products improve host gut environment, microbial balance, and immune system, reduce stress response, synthesize vitamins, decrease pH, release bacteriocin, and have antimicrobial activity (9–14). Using probiotic, phytobiotic, pre-biotic, spirulina, symbiotic, and their combination as feed supplement will open a new door to safe poultry production and economic benefits for farmers and public health (8, 15). Recently, phytogenic feed additives have been successfully used as growth promoter alternatives in feed antibiotics due to their positive impact on growth and the immune system and reduced stress response (16). As any of these natural in-feed additives perform better than AGPs without any side effects, here we desired to increase the usage of these worthy components in order to reduce and overcome the overuse of antibiotics—to be more specific, for the reduction of AMR and for the sustainable safety of poultry production. Therefore, in this article, our motive is to introduce the extensive advantages of probiotic, pre-biotic, phytobiotic, spirulina, symbiotic, and some of their combinations as growth enhancer alternatives to antibiotics. We believed that this review article would definitely be helpful to researchers, pharmacists, veterinarians, the pharmaceutical industry, and poultry producers as a perspective on safe poultry production.

## Probiotics

According to FAO/WHO (17), probiotics are “live microorganisms which, when administered in adequate amounts, confer a health benefit on the host.” Lilley and Stillwell (18) first introduced the term “probiotic” to describe the “growth-promoting factors” produced by microorganisms. Probiotics (i) improve gut health, inhibit the growth of pathogens, and reduce mortality; (ii) maintain the equilibrium in gut microbial population, which improves intestinal ecology; (iii) reduce the incidence of diarrhea by preventing digestive upset; (iv) increase the growth rate, weight gain, and productivity by improving fed intake and conversion efficiency; (v) enhance the activity of digestive enzymes, which increases nutrient absorption and reduces plasma cholesterol by regulating lipid metabolism; (vi) synthesize vitamin B complex and produce short-chain fatty acid in the intestine, which could alter the microbial composition in the gut; (vii) reduce stress related to vaccination, antibiotic therapy, temperature, transportation, etc.; (viii) enhance the efficacy of vaccines and help in faster detoxification of mycotoxins; (ix) improve fertility and egg quality, reduce chick mortality, and leave no residues in products; (x) reduce ammonia and fecal water contents, which improves litter quality and decreases environmental pollution; and (xi) are cost-effective, prevent the development of antimicrobial resistant bacteria, and help in the production of safe food for consumers (15, 19). Certain non-pathogenic species of bacteria, fungi, yeasts, or their combination have been used as probiotics. Probiotics can be classified into colonizing species (*Lactobacillus* sp. *Enterococcus* sp., and *Streptococcus* sp.) and free, non-colonizing species (*Bacillus* sp. and *Saccharomyces cerevisiae*). The microbes generally used to develop probiotics are *Lactobacillus acidophilus, L. sporogenes, L. bulgaricus, L. casei, L. plantarum, L. cellobiosus, L. salivarius, Streptococcus faecium, S. thermophiles, Bacillus coagulans, B. licheniformis, Bifidobacterium bifidum, Saccharomyces cerevisiae, Enterococcus faecium, Torulopsis* sp., and *Aspergillus oryza*, which have beneficial effects on broiler performance (15, 20). Probiotics help the elimination of stress-induced anomalies in the gastrointestinal system, hence restoring normal gut activity (21). Probiotics favor in the production of lactic acid and hydrogen peroxide which are harmful to many pathogens, lowering the oxidation–reduction potential in the gut, which inhibits aerobic pathogens such as *Staphylococcus aureus, Escherichia coli, Salmonella Enteritidis, S. Typhimurium, Clostridium perfringens, Listeria monocytogenes, Campylobacter jejuni, Yersinia enterocolitica, Candida albicans*, and the coccidian parasites *Eimeria* sp. (15, 22). The mode of action of probiotics include alteration in intestinal flora, enhancement of growth of non-pathogenic facultative anaerobic and Gram-positive bacteria, suppression of growth of intestinal pathogens, and improvement of digestion and utilization of nutrients (23). The antimicrobial activity of *Lactobacillus* strains, *B. longum*, and *L. johnsonii* was observed against *Salmonella, Enteritidis*, and *Listeria monocytogenes, Campylobacter* species, and *Clostridium perfringens*, respectively (15). Probiotic and competitive exclusion approach methods have been used to control endemic and zoonotic diseases in poultry. Exclusion of competition represents competition for binding sites on the intestinal mucous membranes and thus prevents pathogenic microorganisms from colonizing the digestive tract, and the third way is competition for nutrients (24). Besides these, probiotics also contribute in the improvement of the health status of bird by reducing toxic amine and ammonia accumulation, production of essential digestive enzymes, production of B-complex vitamins, and appetite stimulation (8, 25). Kabir et al. (26) observed significant body weight gain and higher antibody production in vaccinated and non-vaccinated birds fed with probiotics time dependently. Similarly, Khaksefidi and Ghoorchi (27) indicated that the antibody titer in the 50-mg/kg-probiotic-fed group was significantly higher at 5 and 10 days post-immunization compared to the control when sheep red blood cell was injected at 7 and 14 days of age. In addition, Mahajan et al. (28) observed a lower total viable count in the meat of birds when supplemented with probiotic (Lacto-Sacc) as compared to the meat obtained from control birds. On the other hand, Zhang et al. (29) reported that meat tenderness was improved by supplementation with whole yeast or *Saccharomyces cerevisiae* extract in bird feed. Alam and Ferdaushi (7) reported that probiotic supplementation significantly increased dressed carcass weight, abdominal fat, and breast, thigh, and liver and lower abdominal fat percentage in broiler chicks up to 28 days. A previous report also indicated that the use of probiotics (*L. acidophilus* and *S. faecium*) increased moisture, protein, ash, water holding capacity, emulsion capacity, and stability in broiler meats (28). *B. licheniformis* was also found to have a beneficial effect in improving broiler meat quality (30). Georgieva et al. (31) evaluated a significant feed conversion ratio at the 49th day of age in broiler chicken when supplemented with commercial probiotics (Lacto-Saccaro). Recently Hussein et al. (32) reported that supplementing broiler feed with probiotics and photobiotics, alone or in combination, improves performance and intestinal health in broiler chicks. According to Lukic et al. (33), multi-strain probiotics have a beneficial effect on the host by increasing growth-promoting bacteria, combined with the viable antibiosis of pathogenic bacteria in the intestinal tract. In addition, According to Patel et al. (34), probiotics (Protexin) at 100 g/ton in feed significantly boosted body weight gain and FCR, with no negative effects on feed intake, motility, or carcass characteristics. Moreover, adding probiotics in the feed of laying chickens also enhanced the egg production and improved the egg quality (35). Finally, a sub-therapeutic level of antibiotics used in broiler feed could be replaced by probiotics, as was also reported by Palod and Singh (36).

## Prebiotics

In 1995, Gibson and Roberfroid (37) first introduced pre-biotic as a non-digestible food element that improves the microbial balance of the host by selectively stimulating the growth of and/or activating the metabolism of one or a small number of health-promoting bacteria in the digestive tract. They are consisting of short-chain carbohydrates, principally oligosaccharides, such as fructo-oligosaccharides, galacto-oligosaccharides, and inulin (37, 38). A good pre-biotic should be processed easily in a large scale, always palatable as a feed ingredient, which induces systemic effects to improve host health, and must not be hydrolyzed or absorbed in the upper part of the gastrointestinal (GI) tract (19). Prebiotics (i) provide a substrate to beneficial intestinal microorganisms to accelerate the growth rate and/or proliferation; (ii) alter the GI microflora, stimulate the immune system, reduce pathogen invasion, and reduce cholesterol and odor compounds; (iii) improve gut health by balancing the intestinal microbes, promoting enzyme reaction, and reducing products of phenol and ammonia; and (iv) reduce the production cost (19, 39–42). Prebiotics are capable of enhancing the growth of beneficial bacteria, such as *Bifidobacterium* and *Lactobacillus*, but not pathogens causing gastrointestinal diseases such as *C. perfringens* (37, 43). Fallah and Rezaei (44) reported that the addition of pre-biotic supplementations to broiler diets improved the growth performance and carcass characteristics and decreased the serum cholesterol level of the broiler chickens at 42 days of age. Prebiotics can be used as a potential alternative to growth-promoting antibiotic by altering the intestinal microbes and the immune system to reduce colonization by pathogens, by enhancing nutrient utilization (amino acid and protein), by improving gut health, and also by improving performance (45). Scholz-Ahrens et al. (46) observed a significant increase in the bioavailability of minerals in the gut. Houshmand et al. (47) observed that prebiotics influenced performance and stocking density, resulting in better feed conversion ratio. In addition, Patterson and Burkholder (48) have reported that pre-biotic supplementation has been shown to improve the health status of the gastrointestinal tract in birds. Recently, Froebel et al. (49) have shown that prebiotics enhance growth performance and reduce human foodborne pathogens in poultry.

## Phytobiotics

Phytobiotics are secondary plant metabolites which are natural, less toxic, residue-free, and growth-enhancing feed additives. These are also called phytogenics or botanicals that are composed of natural bioactive substances of plant origin, including alkaloids, glycosides, terpenoids, and phenolics (50). Phytobiotics could be classified as herbs from flowering, non-persistent, and non-woody botanicals or spices from non-leaf parts like fruits, seed, bark, or root, essential oils or extracts, and oleoresins (51). Phytobiotics (i) modify the gut microflora by reducing the number of pathogenic organisms; (ii) improve the performance and digestibility by stimulating salivation, secretion of digestible enzymes, and bile production; (iii) improve the normal intestinal architecture, increase the villus length, and also increase the intestinal surface absorption; and (iv) stimulate the secretion of high amounts of intestinal mucus, consequently alleviating pathogen adhesion and establishing gut microbial eubiosis (52–54). Cross et al. (55) found that some plant extracts enhance the digestion and secretion of digestive enzymes and also exhibit antibacterial, antiviral, and antioxidant properties. Supplementing broiler diet with clove and cinnamaldehyde (56), garlic (57), a mixture of garlic, mushroom, and propolis (58), essential oil mixtures of thymol and star anise (59), oregano (60), a blend of carvacrol, cinnamaldehyde, and capsicum oleoresin (61), and ginger extract (62) had been found to enhance the performance parameters, including feed consumption, FCR, and body weight. Due to the high antibacterial activity of medium-chain fatty acids, nutritional antibiotic can be replaced with phytobiotic. Ripon et al. (6) used Galibiotic in broiler feed and found that phytobiotics (Galibiotic at 10 gm/kg) significantly lowered the intestinal pH, total viable count, and total coliform count, with best growth performance. Simultaneously, phytobiotics in broiler diet reduce crop and cecal pH (63). A low pH increases the growth of beneficial microorganism, improves nutrient absorption, and thus improves growth performance (64). On the other hand, phytochemical compounds of phytobiotics act against Gram-positive and Gram-negative bacteria either in *in vivo* (64, 65) or in *in vitro* environment (66) and were also found to reduce the severity of *Eimeria* spp. infection in broilers by alleviation of dropping score and intestinal lesion score and also reducing oocyst shedding (67). Besides these, essential oils of lemon, green tea, and turmeric blend have a great efficacy in reducing the count of *S. enteritidis* and *Campylobacter jejuni* on the carcass surface of chicken (68). The Eucalyptus volatile oils also have the ability to relieve broilers from complicated respiratory distress caused by *Mycoplasma gallisepticum* (69). In addition, an immunomodulatory effect is also induced by phytobiotics through increasing immune cell proliferation, increasing cytokine expression, and elevation of antibody titer (70, 71).

## Spirulina

Spirulina refers to common blue-green algae that are a natural source of protein, essential amino acids, essential fatty acids, and minerals (72, 73). It is also rich in thiamin, riboflavin, pyridoxin, vitamin B12, vitamin C, and antioxidant carotenoids and is commonly used as a feed component in broiler and layer diets throughout the world (74). Spirulina (i) increases good growth and feed efficiency when added to chick and broiler diet, (ii) induces the growth of beneficial bacteria in the gut which improve broiler health, (iii) upregulates macrophage phagocytic as well as metabolic pathways, (iv) increases disease resistance potential in chicken, (v) acts as natural color enhancers in meats and eggs, and (vi) enriches meat and eggs with polyunsaturated fatty acids (75). Spirulina helps to improve mineral absorption and nutrient digestion processes and protects the gut from diarrhea (76). Spirulina has antiviral activity and immune stimulatory effects (77). Improvement of performance parameters, like FCR, body weight gain, greater production, and percentage of carcass yield, was observed in the dietary group supplemented with *Spirulina platensis* compared with the other groups (78–83). On the other hand, Saxena et al. (84), Venkataraman et al. (85), and Fathi et al. (86) used different concentrations of spirulina in feed and reported that there was no adverse effect on the performance of chickens and thus could be used as a substitute of other feed additives. Access to spirulina powder in the diet at a level of 1% or in drinking water at 0.25% in Japanese quails had beneficial effects on body weight, weight gain, FCR, and fertility when compared with the control groups (87). The final body weight, weekly body weight gain, and FCR of broilers were significantly higher when fed on 6% *Spirulina platensis* than those of birds fed on 3% of these algae at 42 days of age (88). Considering egg production, it was shown that the diet with spirulina (1.5, 3.0, 6.0, or 12.0%) of laying birds increases egg production with good quality (74, 78). Spirulina is a good source of pigments like carotenoids and xanthophylls, which improve the intensity of egg yolk color; in quails fed with 1% dietary spirulina, an optimal color of the egg yolk was induced (89). A recent study found that the boiled eggs of the spirulina-treated group exhibited a more accepted yolk color than the control group (90). Ginzberg et al. (91) studied the effect of microalgae on the fatty acid content of eggs and found that spirulina-treated eggs have decreased contents of cholesterol and saturated fatty acid, thus increasing the level of omega-3 polysaturated fatty acids. Layer chicken, with ages of 29 to 40 weeks, supplied with 3 g spirulina/kg feed had a significantly higher egg production, egg weight, and egg mass compared to the control group (92). Less than 1% spirulina added to chicken diets enhanced the defense systems for antigen processing, greater T-cell activity, and increased microbial killing (93). Supplementation of dietary spirulina improves the health conditions, indicating the enhancement of the immune response against diseases (94, 95). Moreover, a supply of spirulina at the level of 0.05% to broiler ration could particularly reduce the adverse effect of mycotoxin on the weight of bursa, thymus, and spleen (96). Previous studies also demonstrated that treatment with spirulina increased the number of leukocytes and recommended such as a feed additive to increase the immunity of infected chicken against the avian influenza (AI) H5N1 virus (97). *Spirulina platensis* (0.5 and 1 g/kg) and vitamin E (75 mg/kg) in the diet enhanced the total antibody production specific for new castle disease virus (98). Shinde et al. (99) conducted an experiment and reported that the inclusion of 0.06% of spirulina in broiler diet as an herbal feed additive is beneficial in improving the feed consumption and feed conversion efficiency. Our recent studies have shown that spirulina (4 gm spirulina/kg layer feed) has a positive effect on egg production in layer birds, with significantly higher (*P* < 0.05) total erythrocyte count, hemoglobin concentration, and packed cell volume compared to the control group. Serum total cholesterol and triglyceride were significantly decreased in the spirulina-treated group compared to the control group, whereas the high-density lipoprotein cholesterol was increased (*P* < 0.01) in the treated group. Finally, our present work explores the prospective use of spirulina to improve the lipid profile, hematological parameters, and egg production in layer birds (100).

## Synbiotics

Synbiotics are a mixture of probiotics and prebiotics which ensure the growth of probiotics (101, 102). Synbiotics provide a live culture and feed them to improve the survival, persistence, and better growth of useful organisms in the gut of birds as the specific substrate for fermentation (101–103). Fructo-oligosaccharides and bifidobacteria as well as lactitol and lactobacilli are the commonly known combinations of probiotics and prebiotics for use as synbiotics (101). The intestinal microflora plays a crucial role in bird health, and if the equilibrium between beneficial microbes is disrupted, the health and general performance of a bird will be affected. This necessitates further investigation of the effect of food supplementation in the form of prebiotics, which can be supplied to stimulate the establishment of beneficial bacteria in chickens, hence increasing the production. In the diet of chickens, synbiotics were effective in increasing broiler growth (104, 105). Synbiotics were observed to improve the intestinal morphology and the level of nutritional absorption in broiler chickens, resulting in improved performance (106, 107). Only a few research have reported the best ways to use synbiotics in poultry (108). Finding the ideal combination of probiotic and pre-biotic as well as evaluating their synergistic effects for use as prospective synbiotics to maintain proper health requires a lot of attention. Madej et al. (109) found that an *in vivo* injection of inulin (pre-biotic) combined with the *Lactobacillus* bacteria altered the development of numerous immunological organs in broilers.

The results from another study showed that the use of synbiotic (Gallipro + immunoval) in poultry diets significantly improved the body weight gain and FCR (110). Panda et al. (111) found that, during the trial period (1–42 days), chicks whose meals included *L. sporogenes* (as probiotics) gained more daily weight and had a better FCR. Prebiotics such as fructo-oligosaccharides and mannan oligosaccharide have also been shown to boost poultry performance (101, 112). Prebiotics may boost probiotic strain development and cloning; therefore, a probiotic-plus-pre-biotic composition may have more benefits than either of them alone. In addition, Mokhtari et al. (110) also reported that the improvement in growth performance observed in symbiotic treatment can be a proof of this assertion. According to the results obtained by Mokhtri et al. (110), it was found to have many benefits for broiler production by adding various growth stimuli. It is clear that adding synbiotics to poultry diets caused a positive effect on performance and the carcass weight produced (110). In a separate study, Al-Sultan et al. (113) found that, in comparison to prebiotics and organic acids, the symbiotic-supplemented group followed by probiotic-supplemented group had the highest final body weight and time-dependent weight gain, better FCR, and the strongest antibody response to Newcastle disease. Furthermore, when compared to prebiotics and organic acids, synbiotics followed by probiotic treatment enhanced the gut morphology and microbial ecology significantly. Finally, when comparing prebiotics and organic acids as effective growth and health boosters for broilers, it may be proposed that using synbiotics followed by probiotics is preferred.

## Combination of Probiotics and Phytobiotics

The combination of probiotics and phytobiotics can have beneficial synergistic effects on the gut microbiota in young chickens (114). In addition, when used in broiler feeding, the combination of probiotics and phytobiotics can improve production performance, FCR, gut ecology, immunity, antioxidant status, and also the quality of meat from broilers (8, 115). In our previous study, the combined use of probiotics and phytobiotics in broiler feed showed a significant influence on growth performance, carcass performance, and the amount of stripping with the highest packed cell volume, hemoglobin concentration, visceral organ weight, and increased total viable count after 28 days (8). Increased body weight and better feed conversion ratio due to the combined use of probiotic and phytobiotics in broiler feeds were also observed in many studies (116–118). The probiotics and phytobiotics together promote better intestinal growth and provide more energy for the growth of beneficial bacteria in the gut (114, 119). Increased broiler chicken immunity against *Salmonella, E. coli*, and *C. perfringens* infections was also reported in many studies (120, 121). The growth of pathogenic bacteria (*E. coli* and *C. perfringens*) in the intestines of broiler chickens was also inhibited (8, 32, 122, 123). The combined probiotics produce lower abdominal fat and reduce the mass of meat cholesterol with increased total phenols, flavonoids, and antioxidant activity of broiler chicken meat (118, 124).

## Conclusion

Along with our recently published data and evidences published elsewhere, the use of probiotics, prebiotics, phytobiotics, spirulina, synbiotics, and combinations of probiotic and phytobiotics as growth enhancers/feed additives have many potential benefits, including improved digestion and absorption of nutrients, optimization of feed conversion ratio, growth performance, immunomodulation, and improved gut function and health through the exclusion and inhibition of pathogens in the intestine; thereby, the level of safety of poultry products for human consumption is being improved. However, their effectiveness as growth enhancers is dependent on a number of circumstances, including diet compatibility and the alternatives used, hygiene standards, and sound farm management practices. In this regard, more research is needed to look at other combinations of these alternatives with a specific target relationship between the host and beneficial bacteria as well as a more precise recommended dose to boost layer and broiler production. Furthermore, in light of customer demand for safe meat and eggs as well as attempts to prevent antimicrobial resistance, more options for the use of antibiotics as growth enhancer in poultry production with desirable attributes without compromising the welfare of layer and broiler birds must be explored. Meanwhile, awareness must be raised to prevent the irrational use of antibiotics as an in-feed growth booster or for the prevention of diseases in the poultry industry.

## Author Contributions

KR and MT revised and finalized the manuscript. RA, MMH, and RI prepare the draft. MIH, SS, and MI revised and edit the reference section. All authors contributed to the article and approved the submitted version.

## Conflict of Interest

The authors declare that the research was conducted in the absence of any commercial or financial relationships that could be construed as a potential conflict of interest.

## Publisher's Note

All claims expressed in this article are solely those of the authors and do not necessarily represent those of their affiliated organizations, or those of the publisher, the editors and the reviewers. Any product that may be evaluated in this article, or claim that may be made by its manufacturer, is not guaranteed or endorsed by the publisher.
